# Exoscope-Associated Upper-Limb Rather than Neck Postural Changes During Frontal Sinus Procedures in Bifrontal Craniotomies

**DOI:** 10.3390/brainsci16090954

**Published:** 2026-09-07

**Authors:** Miku Tsuruya, Fumihiro Matano, Hiroyuki Dan, Satsuki Imaoka, Megumi Tomita, Kaoru Namatame, Yohei Nounaka, Takao Kitamura, Kazutaka Shirokane, Katsuya Umeoka, Yasuo Murai

**Affiliations:** 1 Department of Neurological Surgery, Nippon Medical School Hospital, 1-1-5 Sendagi, Bunkyo-ku, Tokyo 113-8603, Japan; s00-078@nms.ac.jp (F.M.); h-dan@nms.ac.jp (H.D.); 17satsuki.i.01@nms.ac.jp (S.I.); y-nonaka@nms.ac.jp (Y.N.);; 2 Department of Neurosurgery, Nippon Medical School Tama Nagayama Hospital, 1-7-1 Nagayama, Tama, Tokyo 206-8512, Japan

**Keywords:** microsurgery, exoscopic surgery, frontal sinus procedures, bifrontal craniotomy, surgical ergonomics

## Abstract

**Highlights:**

**What are the main findings?**

- Exoscope use was associated with reduced upper arm elevation and an increased elbow angle.
- No clear change was observed in the neck angle or the RULA-inspired neck score.

**What are the implications of the main findings?**

- Reduced upper arm and shoulder elevation may have implications for cervicoscapular muscular load or fatigue despite no clear change in the static two-dimensional neck angle.
- Whether these postural changes translate into reduced muscular load or symptoms requires direct muscle-activity and symptom-based evaluation.

**Abstract:**

**Objectives:** Exoscopes decouple viewing from eyepieces and may improve surgeon ergonomics. We compared upper-limb and neck posture during microscope- and exoscope-assisted frontal sinus procedures in bifrontal craniotomies. **Methods:** Five surgical cases involving five surgeons yielded 12 paired microscope–exoscope posture observations. Eight pairs from four cases met image-selection criteria and underwent angular measurement using ImageJ version 1.54g and RULA-inspired partial postural assessment. **Results:** Upper arm elevation decreased from 67.3 ± 6.9° under the microscope to 53.5 ± 5.2° under the exoscope (mean change [exoscope minus microscope], −13.8°; 95% CI, −18.0 to −9.6°; *p* = 0.0001). Elbow angle increased from 36.1 ± 16.6° to 64.4 ± 8.0° (mean change, +28.3°; 95% CI, +15.5 to +41.2°; *p* = 0.0012). Neck angle showed no clear change (12.3 ± 4.0° vs. 12.9 ± 7.2°; mean change, +0.6°; 95% CI, −4.1 to +5.4°; *p* = 0.7666). The upper-arm-related score, including shoulder-elevation and abduction points, was lower under the exoscope (*p* = 0.0156), whereas the forearm score was numerically lower (*p* = 0.1250). **Conclusions:** In this exploratory study, exoscope use was associated with reduced upper arm elevation and an increased elbow angle, but no clear change in the neck angle. The predominance and consistency of the upper-limb findings raise the hypothesis that exoscopic visualization may lessen cervicoscapular muscular demand through reduced upper arm and shoulder elevation, even without a measurable change in the static two-dimensional neck angle. Larger studies incorporating three-dimensional motion analysis, muscle activity, fatigue assessment, and clinical outcome measures are needed to determine whether these postural differences translate into reduced musculoskeletal load or injury risk.

## 1. Introduction

Surgical visualization devices are a fundamental component of neurosurgery. Since their introduction in the 1960s, surgical microscopes have been widely used as essential devices that provide high magnification, intense illumination, and stereoscopic visualization, thereby contributing substantially to advances in neurosurgical procedures [1].

At the same time, musculoskeletal disorders associated with prolonged surgery represent an important occupational issue for surgeons. Surveys of surgeons have reported a high prevalence of musculoskeletal symptoms in the neck and shoulder regions, and sustained static or awkward postures may contribute to these symptoms. In some cases, occupational musculoskeletal injury has also been associated with a reduction in operative volume during recovery and perceived effects on surgical performance. Therefore, improving surgeon ergonomics is important not only for the surgeon’s own health, but also for maintaining stable operative performance [2,3].

In recent years, exoscopes have been introduced as next-generation visualization systems, and reports on their clinical application have been increasing. In addition to providing good visibility and educational utility, exoscopes have been reported to potentially improve surgeon ergonomics [4,5,6].

Beyond its role as an alternative visualization device, the exoscope represents part of a broader transition from eyepiece-dependent optical microscopy to monitor-based surgery [7,8]. Its compact design, flexible working distance and viewing direction, and screen-based visualization may increase freedom in surgeon positioning while facilitating shared visualization of the operative field for surgical training and team participation [7]. Recent clinical experience has further emphasized the value of high-definition 4K-3D visualization not only for the primary surgeon but also for assistants and other operating-room personnel, facilitating team collaboration and surgical education [9]. Robotic arm-enabled exoscopes have expanded this role by providing multiscreen output and flexible camera adjustment through foot- and voice-activated controls across a range of cranial and spinal procedures [10]. More recently, exoscopic and hybrid digital visualization systems have begun incorporating automated camera-centering and other digitally assisted functions intended to improve intraoperative workflow [11]. Thus, the role of the exoscope is expanding from magnification and illumination alone toward shared visualization, education, team collaboration, and digitally assisted surgical workflows. As the range of exoscope-assisted procedures expands, procedure-specific evaluation of surgeon posture and physical demands becomes increasingly relevant.

In microscope-assisted surgery, the surgeon must continuously look through the ocular eyepieces, which restricts the freedom of the visual axis and may force a nonphysiological posture depending on the surgical target and direction of manipulation [12,13]. In particular, procedures requiring visualization of deep or cranially located operative fields may require the surgeon to maintain a posture that places strain on the neck, shoulder, and upper limb. In contrast, with the exoscope, the surgeon is freed from the ocular eyepieces and observes the operative field through a monitor. This allows the observation axis to be separated from the surgeon’s viewing direction and working posture. Through this feature, the exoscope may reduce postural strain and provide visualization from angles that are difficult to obtain using a conventional microscope [4,14].

However, the exoscope is not considered suitable for all procedures. Previous reports have suggested that the microscope may be advantageous in narrow operative fields or surgical corridors, in procedures requiring particularly precise discrimination of color tone or fine structures, or in cases that require frequent camera adjustment [4,5]. Therefore, to evaluate the utility of the exoscope, it is important to clarify in which types of procedures its advantages are most likely to be demonstrated.

Frontal sinus procedures performed during anterior skull base surgery or bifrontal craniotomy are among the surgical steps in which conventional microscope-assisted manipulation may impose substantial postural strain. Because the operative field is located cranially and anteriorly, the surgeon often works along a visual axis directed upward. The exoscope may increase the freedom of head, neck, and upper limb posture and thereby reduce conventional postural strain.

Although ergonomic advantages of exoscopic surgery have been reported [4,5,12], few studies have quantitatively compared surgeon posture under the microscope and exoscope in specific procedures that require upward visualization and are susceptible to visual-axis constraints, such as frontal sinus procedures. In addition, it remains unclear which body regions show measurable exoscope-associated postural differences. Therefore, we quantitatively compared surgeon posture during microscope-assisted and exoscope-assisted frontal sinus procedures in bifrontal craniotomies using intraoperative photographs. The aim of this study was to quantitatively compare upper-limb and neck posture between microscopic and exoscopic visualization during this surgical step and to explore the postural aspects of surgeon ergonomics associated with the two visualization systems.

## 2. Materials and Methods

### 2.1. Study Design and Patients

To explore the potential of the exoscope for frontal sinus procedures, we conducted a single-center retrospective observational study comparing surgeon posture during microscope-assisted and exoscope-assisted procedures.

We reviewed five surgical cases of anterior skull base surgery or bifrontal craniotomy requiring frontal sinus procedures performed at our institution between May 2025 and May 2026. Microscope and exoscope posture images obtained for a single surgeon within the same surgical case were defined as one observation pair. A total of 12 candidate observation pairs were obtained from five surgeons participating in the five surgical cases. Surgeon characteristics, including sex, height, usual operative role, and number of analyzed observation pairs, are presented in Table 1. Because the numbers of surgeons and observations were limited, comparisons according to surgeon characteristics were not performed; instead, these characteristics were treated as descriptive information relevant to the interpretation of the results.

**Figure 1:**
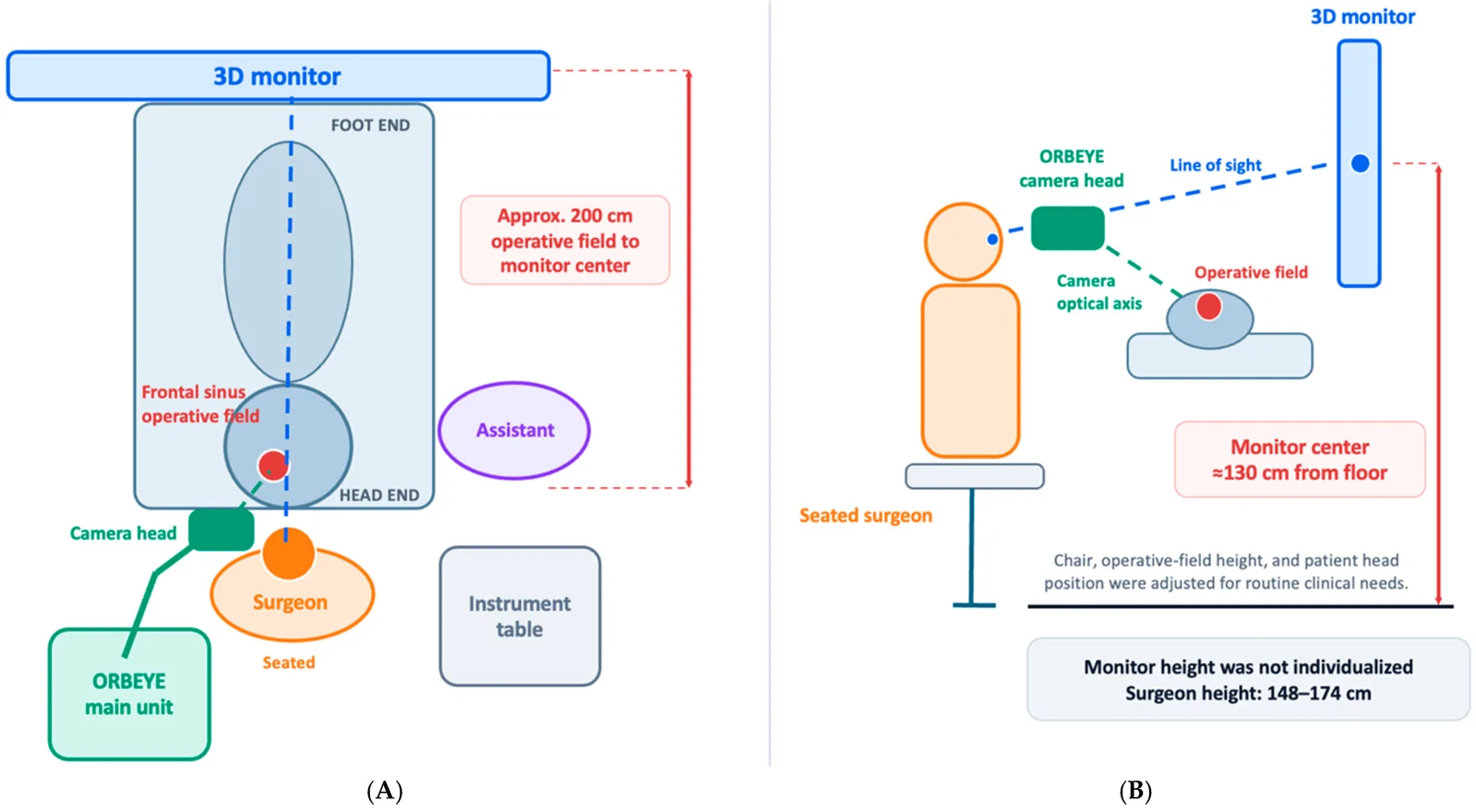
Basic operating room setup during exoscope-assisted procedures. Schematic operating-room setup and viewing geometry during exoscope-assisted frontal sinus procedures. (**A**) Top view of the operating-room layout. The ORBEYE main unit was positioned behind and to the left of the seated surgeon, with the camera head directed toward the frontal sinus operative field. The 3D monitor was positioned at the foot end of the operating table. The blue dashed line represents the surgeon’s line of sight toward the monitor, and the green dashed line represents the ORBEYE camera optical axis toward the operative field. The horizontal distance from the frontal sinus operative field to the center of the monitor was approximately 200 cm. (**B**) Side view of the monitor and surgeon. The center of the monitor was positioned approximately 130 cm above the floor and was not individually adjusted for surgeons whose heights ranged from 148 to 174 cm. All procedures were performed with the surgeons seated, whereas the chair height, operative-field height, and patient head position were adjusted according to routine clinical needs. The schematic is not drawn to scale.

Both the OPMI PENTERO 900 surgical microscope (Carl Zeiss Meditec AG, Oberkochen, Germany) and the ORBEYE™ exoscope (Olympus, Tokyo, Japan) were used during the frontal sinus procedure in each surgical case. The operating-room setup and the spatial relationships among the surgeon’s line of sight, the ORBEYE camera optical axis, the frontal sinus operative field, and the 3D monitor are shown schematically in Figure 1. The ORBEYE main unit was positioned behind and to the left of the surgeon, and the camera head was adjusted to provide an appropriate viewing axis for the frontal sinus procedure. The 3D monitor was positioned at the foot end of the operating table. In the standard setup, the horizontal distance from the frontal sinus operative field to the center of the monitor screen was approximately 200 cm, and the height from the floor to the center of the monitor was approximately 130 cm. All procedures were performed with the surgeons seated. No dedicated forearm support was used, although the hands could rest near the operative field. The heights of the surgeon’s chair and the operative field and the patient head position could be adjusted for each surgeon and visualization condition according to routine clinical needs; these settings were neither standardized nor quantitatively recorded. The microscope working distance was not recorded. Before image acquisition, a photographic protocol was established in which the camera optical axis was positioned as nearly perpendicular as possible to the plane containing the visible axes of the surgeon’s trunk, upper arm, and forearm. During microscope-assisted and exoscope-assisted manipulation, each surgeon’s posture was photographed from as nearly lateral a direction as possible, and images obtained for the same surgeon within the same surgical case were paired. After image acquisition, two investigators jointly assessed the eligibility of each image according to the predefined protocol. Images obtained from a markedly posterior-oblique or superior-oblique direction, or images in which the relevant body axes could not be defined with sufficient confidence from the visible anatomical contours, were considered unsuitable for angle measurement. Following this eligibility assessment, if multiple microscope or exoscope images remained, two investigators jointly selected the images obtained during the same or most similar procedural step. As a general rule, images were paired when they had been obtained during the same or a similar maneuver immediately before and after a direct switch from the microscope to the exoscope, or vice versa. The analyzed steps mainly involved manipulation within or around the frontal sinus, including drilling or removal of the frontal sinus wall and mucosal handling, depending on the surgical case. All image-pair selections and exclusions were finalized before angle measurement. One of the investigators who participated in image selection subsequently performed the angle measurements. However, the direction and field of photography were subject to certain constraints imposed by the arrangement of equipment in the operating room, the sterile field, and the positions of the surgeon and assistant. The sequence of microscope and exoscope use was not standardized across surgical cases. The case-specific order and duration of use of each visualization system before photography were not recorded at the time of surgery and could not be reliably reconstructed retrospectively.

Four of the 12 candidate observation pairs were excluded before angle measurement because one or both images did not meet the predefined criteria for photographic direction or reliable definition of the relevant body axes. Specifically, three pairs were excluded because the exoscope images had been obtained from a markedly posterior-oblique direction, and the remaining pair was excluded because the exoscope image had been obtained from a markedly superior-oblique direction. In addition, in one of the former three pairs and in the latter pair, visualization of the lower trunk was insufficient to define the trunk axis with sufficient confidence, even after consideration of the surrounding visible body contours. Consequently, the final analysis included eight observation pairs obtained from four surgical cases and five surgeons. All four surgical cases represented in the final analysis involved bifrontal craniotomy.

### 2.2. Angle Measurement Using Intraoperative Images

**Figure 2:**
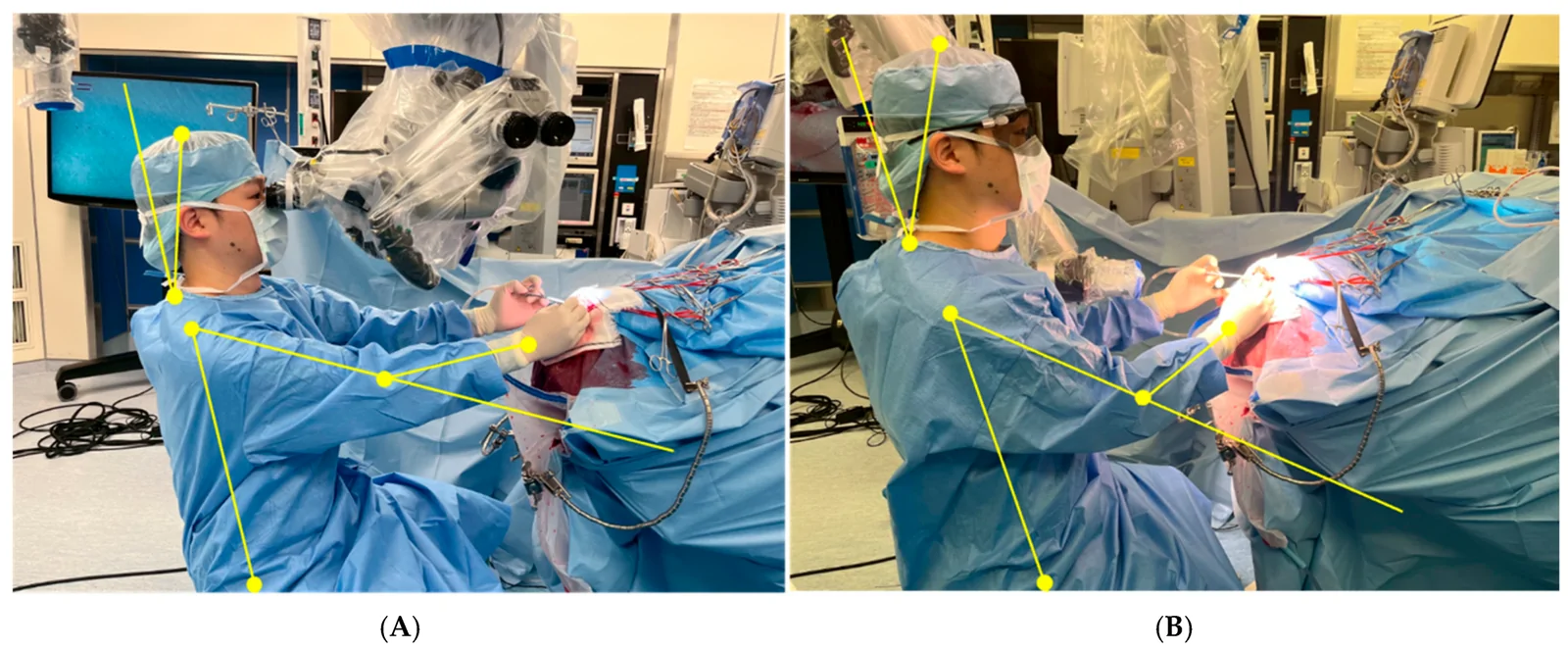

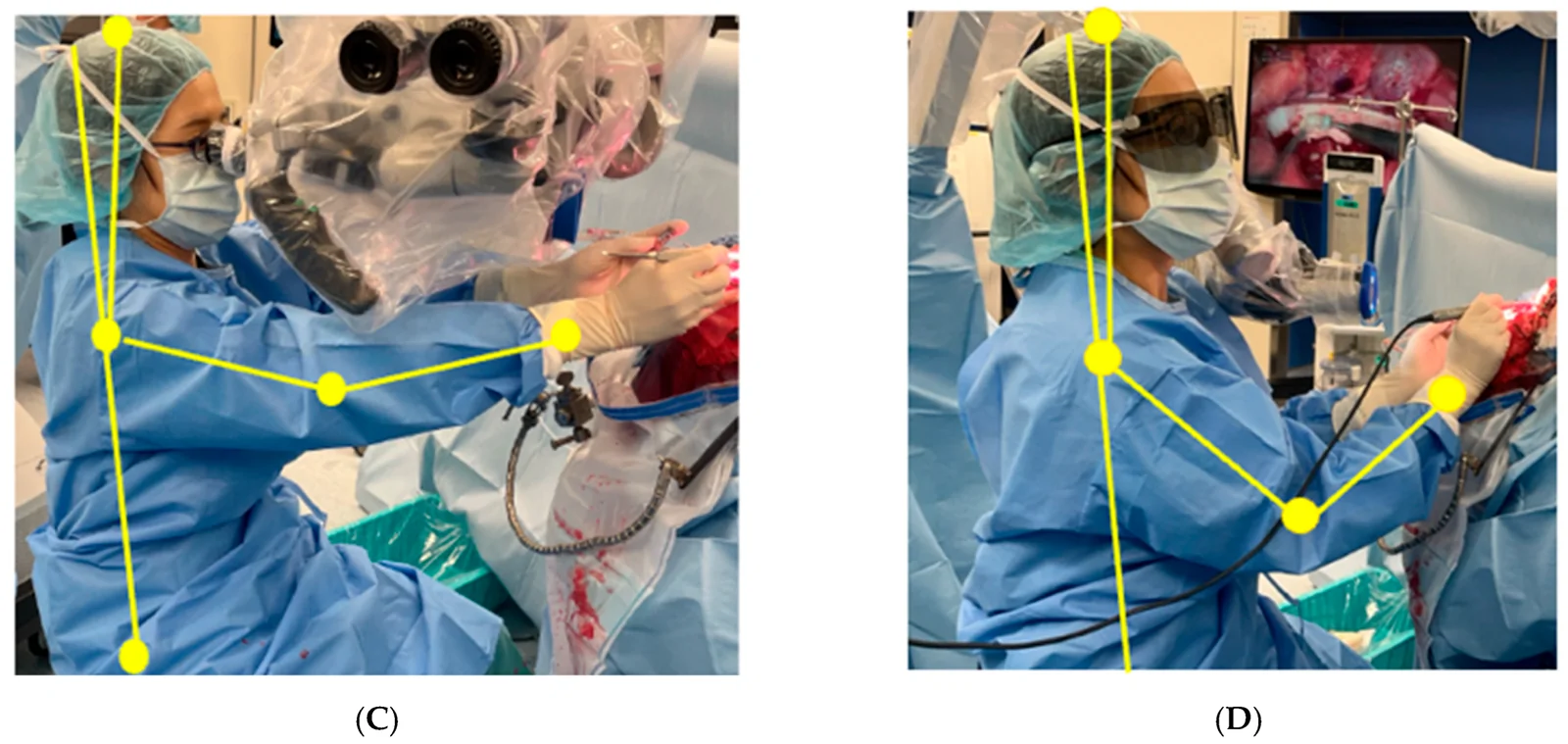
Representative paired intraoperative photographs of surgeon posture under microscopic and exoscopic visualization. (**A**,**B**) Pair 4 and (**C**,**D**) Pair 5. Images within each pair were obtained from the same surgeon, in the same surgical case, and during the same or most similar procedural step. (**A**,**C**) Microscopic visualization; (**B**,**D**) exoscopic visualization. Both pairs showed reduced upper arm and shoulder elevation and an increased elbow angle under exoscopic visualization, whereas the direction of neck-angle change differed between the two pairs. Superimposed lines indicate the body axes used to measure the upper arm elevation, elbow, and neck angles. Partially obscured landmarks or body axes were estimated from the surrounding visible anatomical contours according to the predefined measurement method.

All angle measurements were performed by one examiner using ImageJ version 1.54g (National Institutes of Health, Bethesda, MD, USA). In each observation pair, the upper arm elevation angle, elbow angle, and neck angle were measured two-dimensionally on the paired intraoperative photographs obtained under the microscope and exoscope. Accordingly, all measured angles represented projected angles on the two-dimensional image plane and should not be interpreted as true three-dimensional joint angles. Angle definitions were established with reference to body segment assessments used in the RULA method and in previous surgical posture analyses [15,16]. Two representative microscope–exoscope observation pairs are shown in Figure 2.

The upper arm elevation angle was defined as the angle of the upper arm axis relative to the trunk axis. The upper arm axis was defined as the line connecting the acromion and the olecranon, and the trunk axis was defined as the visible longitudinal axis of the lateral trunk on the photograph. The elbow angle was measured as the angle between the upper arm axis and the forearm axis, with the forearm axis defined as the line connecting the olecranon and the midpoint of the wrist. The neck angle was measured as the angle between the trunk axis and the head-neck axis, with the head-neck axis defined as the line connecting the region around the seventh cervical vertebra (C7) and the vertex of the head. When an anatomical landmark or part of a body axis was partially obscured by surgical clothing, its location or orientation was estimated with reference to the surrounding visible anatomical contours. Images were excluded only when the relevant axis could not be defined with sufficient confidence using this approach. Approximately one month after the initial measurements, the same examiner remeasured all images included in the analysis to assess intra-rater reproducibility. The initial measurements were used for the main analysis, and the repeated measurements were used to evaluate intra-rater error. Intra-rater error was calculated as the absolute difference between the two measurements for each angle. Intra-rater reliability was quantified separately for each angle and visualization condition using a two-way mixed-effects, absolute-agreement, single-measure intraclass correlation coefficient [ICC(A,1)]. Because all measurements were performed on still images, dynamic changes in posture during surgery, rotation of the neck or trunk, and three-dimensional movements of the upper limb were not assessed.

### 2.3. RULA-Inspired Partial Postural Assessment

The results of angle measurement using ImageJ were interpreted with the aid of the Rapid Upper Limb Assessment (RULA)-inspired partial postural assessment. RULA is an observational ergonomic assessment tool used to evaluate postural load, including the upper limbs, neck, trunk, lower limbs, muscle use, and external load [15].

Because the intraoperative photographs used in this study did not consistently include the lower limbs, wrist, or entire trunk, the formal overall RULA score based on the original method was not calculated. Instead, a partial assessment based on the RULA posture criteria was performed for the upper arm, forearm, and neck, which were consistently evaluable on the images. The items used for the assessment are shown in Table 2. For the upper arm, the basic score was assigned according to the upper arm elevation angle, with additional points for shoulder elevation and arm abduction. Shoulder elevation was recorded only when a clear shoulder-shrugging posture was visually evident on the lateral image, with the shoulder contour displaced cranially toward the neck relative to the trunk. In the RULA-inspired forearm posture assessment, an elbow angle of 60–100° was assigned a score of 1, whereas angles outside this range were assigned a score of 2. The additional score based on observation from above the worker could not be assessed and was therefore not applied. The neck was scored based on the measured neck angle.

In this study, these scores were not used as formal RULA grand scores or as primary endpoints. They were used only as RULA-inspired supplementary posture indicators to support the ergonomic interpretation of the measured angles.

### 2.4. Statistical Analysis

Because this study included small numbers of surgical cases, surgeons, and posture-observation pairs, the analyses were performed primarily in a descriptive and exploratory manner. In addition to repeated observations from the same surgeon, the dataset included observations from multiple surgeons within the same surgical case. Thus, the observation pairs were clustered by both surgeon and surgical case and could not be considered fully independent. Because the numbers of surgeons, surgical cases, and observations were limited, stable estimation using mixed-effects models was considered infeasible, and such models were not applied. Therefore, observation-level *p* values were interpreted as exploratory comparative indices rather than confirmatory evidence. A nominal significance threshold of *p* < 0.05 was used for the exploratory analyses.

Measured angles are presented as means ± standard deviations. Before paired *t*-tests were applied, the distributions of the paired differences were inspected for marked asymmetry and extreme outliers; no marked departures were identified. Given the small number of pairs, this assessment was descriptive. Paired *t*-tests were used to compare measured angles between microscope and exoscope use. RULA-inspired partial scores were treated as ordinal variables and are presented as medians [interquartile ranges]. Wilcoxon signed-rank tests were used to compare these scores between microscope and exoscope use. Mean paired changes (exoscope minus microscope) and their 95% confidence intervals were calculated at the observation-pair level. Because these intervals did not account for clustering by surgeon or surgical case, they were interpreted as exploratory estimates of uncertainty.

Statistical analyses were performed using IBM SPSS Statistics version 32.0 (IBM Corp., Armonk, NY, USA). Because of the small number of observations, exact two-sided *p* values were reported for the Wilcoxon signed-rank tests. As descriptive sensitivity analyses addressing clustering, the microscope-to-exoscope change in each measured angle was averaged within each surgeon and separately within each surgical case, yielding one mean change per surgeon (*n* = 5) and one mean change per surgical case (*n* = 4). Exact two-sided Wilcoxon signed-rank tests were applied to these aggregated changes. Given the very small numbers of aggregation units, the results were interpreted primarily according to the magnitude and consistency of the changes rather than statistical significance.

## 3. Results

**Figure 3:**
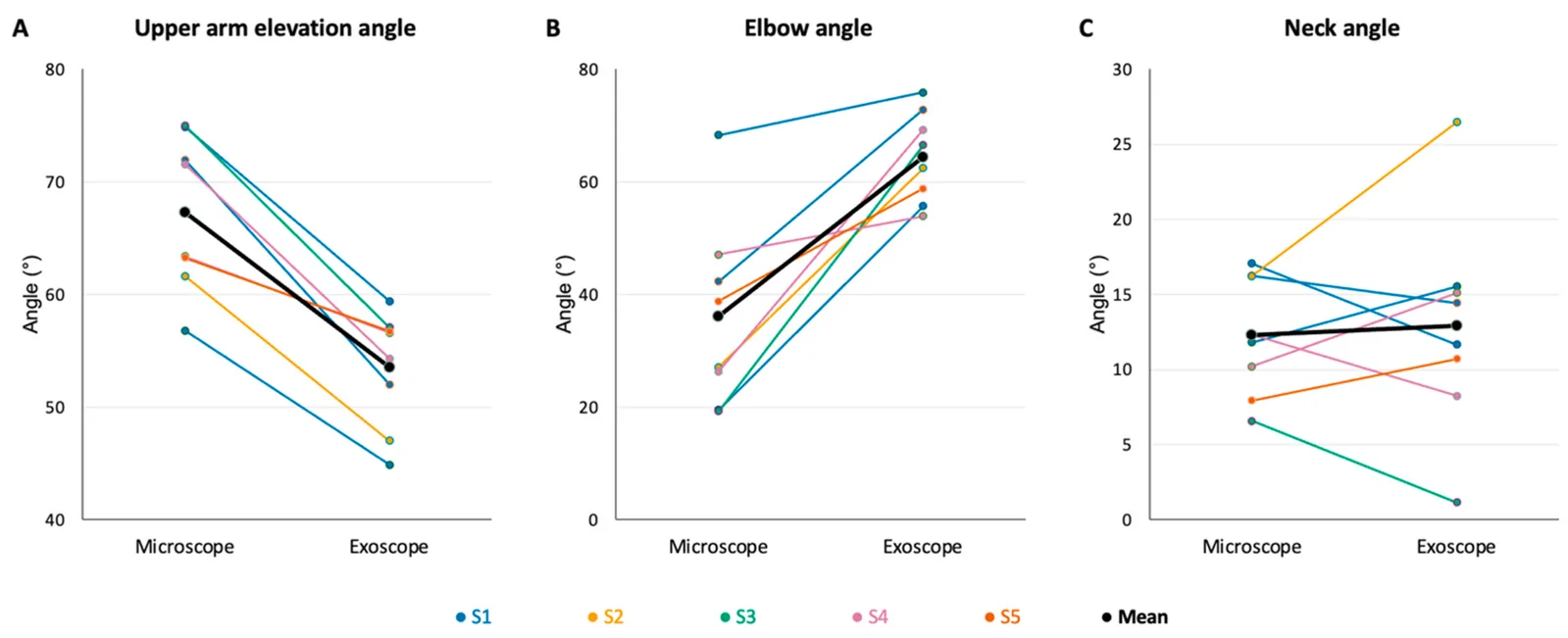
Paired changes in measured angles between microscopic and exoscopic visualization (*n* = 8 paired observations). Note: (**A**) Upper arm elevation angle; (**B**) elbow angle; and (**C**) neck angle. Each thin colored line connects microscope and exoscope measurements from the same observation pair, with colors identifying the five surgeons (S1–S5). The thick black line represents the overall mean. Upper arm elevation decreased and the elbow angle increased in all eight pairs, whereas the direction of neck-angle change varied among pairs.

The measured angles and RULA-inspired partial scores from the main analysis are summarized in Table 3, with the corresponding individual values presented in Table 4. In the assessment of intra-rater error, the absolute differences between the two measurements were 2.1 ± 1.5° for the upper arm elevation angle, 3.1 ± 2.5° for the elbow angle, and 2.3 ± 2.2° for the neck angle. The ICC(A,1) values (95% CIs) under the microscope were 0.933 (0.698–0.986) for upper arm elevation, 0.958 (0.822–0.991) for elbow angle, and 0.863 (0.485–0.971) for neck angle. The corresponding values under the exoscope were 0.823 (0.363–0.962), 0.930 (0.689–0.986), and 0.831 (0.286–0.965), respectively. Point estimates ranged from 0.823 to 0.958, but the confidence intervals were wide, reflecting the small number of observations. At the observation level, the upper arm elevation angle was lower under the exoscope than under the microscope (53.5 ± 5.2° vs. 67.3 ± 6.9°; mean paired change [exoscope minus microscope], −13.8°; 95% CI, −18.0° to −9.6°; *p* = 0.0001). The elbow angle was higher under the exoscope than under the microscope (64.4 ± 8.0° vs. 36.1 ± 16.6°; mean paired change, +28.3°; 95% CI, +15.5° to +41.2°; *p* = 0.0012). In contrast, the neck angle did not differ clearly between the two conditions (12.9 ± 7.2° under the exoscope vs. 12.3 ± 4.0° under the microscope; mean paired change, +0.6°; 95% CI, −4.1° to +5.4°; *p* = 0.7666). These observation-level confidence intervals were exploratory and did not account for clustering by surgeon or surgical case. Figure 3 shows the changes in angles from microscope to exoscope use across the eight posture-observation pairs. The upper arm elevation angle decreased in all eight pairs, and the elbow angle increased in all eight pairs. In contrast, the direction of change in the neck angle varied among observations, with no consistent direction of change. This consistency in the upper-limb measures indicates that the observed mean decrease in upper arm elevation angle and increase in elbow angle were not driven solely by a small number of observation pairs.

In the surgeon-level sensitivity analysis, the mean changes (exoscope minus microscope) were −13.4° for upper arm elevation, +30.4° for elbow angle, and +1.4° for neck angle. Upper arm elevation decreased and elbow angle increased in all five surgeons (exact two-sided Wilcoxon *p* = 0.0625 for each), whereas neck-angle changes were inconsistent (*p* = 0.8125). In the surgical-case-level analysis, the corresponding mean changes were −14.3°, +30.4°, and +1.7°, respectively. Upper arm elevation decreased and elbow angle increased in all four surgical cases (*p* = 0.1250 for each), while neck-angle changes remained inconsistent (*p* = 0.8750). Thus, the surgeon- and surgical-case-level analyses showed the same directional pattern as the observation-level analysis (Table 5).

In the RULA-inspired partial postural assessment, the basic upper arm score was 3 in all observations under both the microscope and the exoscope, and therefore did not distinguish the change in upper arm elevation angle as a categorical score. In contrast, the upper arm-related score including additional points for shoulder elevation and abduction was lower under the exoscope than under the microscope (3 [3–3] vs. 4 [4–4], exploratory *p* = 0.0156). The forearm score was numerically lower under the exoscope than under the microscope (1 [1–2] vs. 2 [2–2]), with no clear between-condition difference (exploratory *p* = 0.1250). The neck score was 2 [1.5–2] under both the microscope and the exoscope, with no clear difference between the two conditions (*p* = 1.000). Figure 2 presents two representative examples corresponding to Pairs 4 and 5 in Table 4. Under exoscopic visualization, the upper arm elevation angle decreased from 61.6° to 47.0° in Pair 4 and from 75.0° to 57.1° in Pair 5. In contrast, the elbow angle increased from 27.1° to 62.4° and from 19.2° to 66.5°, respectively. In both pairs, the elbow angle under exoscopic visualization was within the 60–100° range assigned the lowest forearm posture score in RULA. By contrast, the neck angle increased in Pair 4 but decreased in Pair 5. These representative examples visually illustrate the consistent upper-limb postural changes observed across the dataset—namely, reduced upper arm elevation and increased elbow angle under exoscopic visualization—as well as the inconsistent direction of neck-angle changes. Overall, at the observation level, visualization-device-associated changes were primarily observed in the upper-limb measures, whereas no clear differences were found in the neck angle or RULA-inspired neck score.

## 4. Discussion

In this study, we quantitatively compared surgeon posture during microscope-assisted and exoscope-assisted frontal sinus procedures in bifrontal craniotomies using intraoperative photographs. The exoscope was associated with a decrease in upper arm elevation angle and an increase in elbow angle compared with the microscope. In contrast, no clear difference was observed in the neck angle or the RULA-inspired neck score. These findings indicate that, in this setting, the device-associated postural pattern was concentrated in upper arm and shoulder elevation and elbow position rather than in the neck angle, raising the possibility that this upper-limb and shoulder-girdle pattern may influence cervicoscapular muscular demand despite the absence of a measurable change in the static neck angle.

The same directional pattern was retained in sensitivity analyses that gave equal weight to each surgeon and to each surgical case, indicating that the upper-limb findings were not driven solely by surgeons or surgical cases contributing multiple observation pairs. Nevertheless, because these aggregated analyses included only five surgeons and four surgical cases, they remain descriptive and exploratory. Within these limitations, the concordant direction across observation-, surgeon-, and surgical-case-level analyses supports the internal consistency of the upper-limb pattern in this dataset.

The mean upper arm elevation angle was 67.3° under the microscope and 53.5° under the exoscope (mean paired difference, −13.8°; 95% CI, −18.0° to −9.6°). In the RULA-inspired partial postural assessment, the basic upper arm score was 3 under both conditions and did not distinguish between the two postures. However, the upper arm-related score including additional points for shoulder elevation and abduction was lower under the exoscope (exploratory *p* = 0.0156). In particular, shoulder elevation was observed in 7 of 8 microscopic observations, whereas it was not clearly observed under the exoscope. Thus, a shoulder-shrugging posture was less frequently observed under exoscopic visualization in the analyzed pairs.

A larger change was observed in the elbow angle with exoscope use. The mean elbow angle under the microscope was 36.1°, well below the 60–100° range assigned the lowest forearm posture score in RULA. In contrast, it increased to 64.4° under the exoscope, indicating greater elbow flexion and falling within this range. The RULA-inspired forearm score showed no clear between-condition difference. Although thresholds for practically meaningful changes in these two-dimensional angular measures have not been established, the consistent reduction in upper arm elevation and increase in elbow angle across all observation pairs indicate a reproducible device-associated shift in upper-limb posture. Determining whether this shift translates into reduced muscular load or musculoskeletal risk will require direct physiological and clinical assessment.

During manipulation within the frontal sinus, the operative field is relatively deep and cranially located. Under microscopic visualization, the surgeon must reach the frontal sinus operative field while maintaining eye alignment with the ocular eyepieces, which may require the upper limbs to be extended forward and the elbows to be held in a relatively extended position. In contrast, exoscopic visualization frees the surgeon’s line of sight from the ocular eyepieces, potentially allowing greater flexibility in adjusting the surgeon’s position relative to the operative field and enabling manipulation with greater elbow flexion. Previous studies have also reported postural advantages of exoscopic visualization in surgical settings in which surgeon posture is constrained under microscopic visualization. Our findings are consistent with the possibility that exoscope-associated postural differences may be more apparent during such procedures [6]. In contrast, the mean neck angle was 12.3° under microscopic visualization and 12.9° under exoscopic visualization, with no clear difference between the two conditions. The RULA-inspired neck score showed no clear between-condition difference and was numerically slightly higher under exoscopic visualization. Therefore, our findings do not indicate a reduction in the two-dimensional neck angle measured here. However, this measure was limited to a projected head-neck/trunk angle on static photographs; neck rotation, lateral flexion, duration of posture maintenance, and muscle activity, all of which may contribute to actual neck load, were not assessed. During exoscopic surgery, the surgeon operates while viewing a monitor; therefore, neck posture may be influenced by the height and position of the monitor and its distance from the surgeon. In this study, the heights of the surgeon’s chair and operative field were adjusted for each surgeon, whereas the center of the 3D monitor was positioned at approximately 130 cm above the floor and was not individually adjusted for surgeons whose heights ranged from 148 to 174 cm. Thus, although the relative positions of the surgeon and monitor were adjusted to some extent under routine operative conditions, they were neither standardized nor quantitatively recorded. The lack of a consistent change in neck angle may therefore have been influenced in part by the relative arrangement of the chair, operative field, and monitor. As described above, microscopic visualization requires the surgeon to maintain eye alignment with the ocular eyepieces while viewing the frontal sinus, potentially resulting in a constrained posture involving upper arm elevation, shoulder elevation, and a relatively extended elbow even without a substantial change in neck angle [13]. Upper arm and shoulder elevation are also evaluated as components of upper-limb posture in RULA [15]. Previous ergonomic studies have suggested that sustained activity of the shoulder muscles and trapezius associated with prolonged or awkward postures may contribute to cervicoscapular symptoms [17,18]. Therefore, the reduction in upper arm and shoulder elevation under exoscopic visualization may lessen cervicoscapular muscular demand even without a measurable change in the two-dimensional neck angle. Thus, the absence of a clear change in neck angle does not exclude a potential reduction in neck and shoulder muscular load or fatigue. A recent clinical comparison similarly reported a tendency toward lower fatigue scores with exoscope use despite no significant differences in sensor-measured neck posture [19]. Because muscle activity and subjective fatigue were not assessed, this interpretation remains hypothesis-generating and should be evaluated directly using electromyography and fatigue assessment.

This study has several limitations. First, the final dataset was limited to four surgical cases, five surgeons, and eight posture-observation pairs. In addition to repeated observations from the same surgeon, posture observations from multiple surgeons were obtained within the same surgical case. Thus, the observations were clustered by both surgeon and surgical case and could not be considered fully independent. Therefore, the observation-level analyses and their associated *p* values should be interpreted as exploratory findings. Second, this study used a two-dimensional assessment based on intraoperative still images. All measured angles were projected angles on the image plane and did not represent true three-dimensional joint angles. Although a predefined photographic protocol was used and images with marked obliquity were excluded, photography could not be performed from a fixed and calibrated camera position. Therefore, smaller differences in camera direction and perspective between paired images could not be eliminated. Landmark identification, patient head position, operative-field height relative to the surgeon, the working or focal distance of the visualization device, and case-specific device adjustment may also have affected the observed posture and measured angles. Accordingly, both the absolute angle values and the observed microscope-to-exoscope differences should be interpreted cautiously as two-dimensional observations obtained at selected operative moments. Rotation of the neck or trunk, three-dimensional movements of the upper limb, and dynamic postural changes during surgery could not be evaluated. Third, because of constraints related to the operating room setup, the lower limbs and the entire trunk could not be consistently included in the photographs. Therefore, the formal overall RULA score could not be calculated, and the assessment was limited to partial scoring. Fourth, although surgeon height was recorded, sitting height, upper limb length, and individual arm reach were not directly measured. These body habitus factors, as well as surgical experience, familiarity with the microscope and exoscope, and individual postural habits, may have influenced intraoperative posture and contributed to between-surgeon variability. Fifth, four of the 12 candidate observation pairs were excluded because the photographic direction or the ability to define the relevant body axes with sufficient confidence did not satisfy the predefined criteria. Although all image-pair selections and exclusions were finalized before angle measurement, the examiner who performed the angle measurements also participated in image selection. Therefore, residual subjectivity and selection bias related to image availability and pairing cannot be excluded. Sixth, all angular measurements were performed by a single examiner. Although anatomical landmarks were operationally defined and intra-rater reliability was assessed, inter-rater reliability was not assessed in the present study. Seventh, because the case-specific order of microscope and exoscope use and the duration of use before photography were not recorded, potential confounding by operative progression, fatigue, changes in surgical-field accessibility, and intervening adjustments to patient or equipment position could not be evaluated.

Taken together, in this surgical setting, upper arm elevation was lower and the elbow angle was greater under exoscopic than under microscopic visualization, whereas no consistent change was observed in the neck angle. The concentration and consistency of these findings across observation pairs, surgeons, and surgical cases suggest that the potential ergonomic effects of exoscopic visualization may involve cervicoscapular muscular demand through changes in upper-limb and shoulder-girdle positioning, even when the neck angle remains unchanged. This internal consistency provides a rationale for testing whether reduced upper arm and shoulder elevation is accompanied by lower cervicoscapular muscular load or fatigue; because these outcomes were not measured, this possibility remains hypothetical. Future studies should combine standardized model-based experiments controlling for patient head position, operative-field height, working distance, and monitor position with larger clinical samples incorporating three-dimensional postural analysis, electromyographic assessment of the trapezius and cervical muscles, and subjective assessments of fatigue and discomfort.

## 5. Conclusions

In this study, we compared surgeon posture under microscopic and exoscopic visualization during frontal sinus procedures in bifrontal craniotomies using intraoperative photographs. In this small exploratory sample, exoscope use was associated with a mean 13.8° reduction in upper arm elevation and a mean 28.3° increase in elbow angle, and the directions of these upper-limb changes were consistent across both surgeon- and surgical-case-level sensitivity analyses. In contrast, the mean neck angle changed by only 0.6° and showed no consistent direction. These findings raise the possibility that reductions in upper arm and shoulder elevation under exoscopic visualization may lessen cervicoscapular muscular demand even without a measurable change in the static two-dimensional neck angle. Because muscle activity and fatigue were not assessed, this hypothesis requires direct evaluation in larger studies combining three-dimensional postural analysis, electromyography, fatigue assessment, and clinical outcome measures.

## Figures and Tables

**Table 1:** Characteristics of surgeons included in the postural analysis.

Surgeon ID	Sex	Height, cm	Usual Operative Role	Analyzed Pair ID(s)
S1	Male	171	Senior operator	1, 2, 3
S2	Male	174	Independent operator	4
S3	Female	158	Independent operator	5
S4	Female	153	Supervised operator/first assistant	6, 7
S5	Female	148	Supervised operator/first assistant	8

**Note:** Surgeon IDs were anonymized. Usual operative role was assigned descriptively according to each surgeon’s routine role in cranial neurosurgical procedures: supervised operator/first assistant, usually serving as a first assistant or performing selected surgical steps under supervision; independent operator, regularly serving as the primary surgeon for standard cranial procedures; and senior operator, routinely performing and supervising complex cranial or skull base procedures.

**Table 2:** RULA-inspired partial postural scoring criteria used in this study.

Body Region	Scoring Component	Criterion	Score /Additional Score	Observed Status
Upper arm	Basic upper-arm posture	45–90° flexion	3	All analyzed observations
Upper arm	Shoulder elevation	Clear shoulder-shrugging posture	+1	Observed in 7/8 microscope images and 0/8 exoscope images
Upper arm	Upper arm abduction	Abduction present	+1	Not observed
Forearm	Basic forearm posture	Elbow flexion 60–100° /outside this range	1/2	Both categories observed
Neck	Basic neck posture	0–10°/>10–20°/>20°/extension	1/2/3/4	Scores 1–3 observed; extension not observed

**Note:** Only the components used in the main RULA-inspired partial postural assessment are summarized here. Detailed scoring criteria, including categories considered but not observed or not applied, are provided in Supplementary Table S1. These partial scores do not constitute a formal RULA total/grand score or an estimate of overall musculoskeletal risk.

**Table 3:** Observation-level angular measurements and RULA-inspired partial scores (*n* = 8 paired observations).

Parameter	Microscope	Exoscope	Mean Paired Change (95% CI), °	*p* Value
Upper arm elevation angle	67.3 ± 6.9	53.5 ± 5.2	−13.8 (−18.0 to −9.6)	0.0001
Elbow angle	36.1 ± 16.6	64.4 ± 8.0	+28.3 (+15.5 to +41.2)	0.0012
Neck angle	12.3 ± 4.0	12.9 ± 7.2	+0.6 (−4.1 to +5.4)	0.7666
Upper-arm-related score	4 [4–4]	3 [3–3]	—	0.0156
Forearm score	2 [2–2]	1 [1–2]	—	0.1250
Neck score	1.5 [1–2]	2 [1–2]	—	1.0000

**Note:** Angular measurements are presented as mean ± standard deviation, and RULA-inspired partial scores as median [interquartile range]. Mean paired changes were calculated as exoscope minus microscope. Paired *t*-tests were used for angular measurements, and exact two-sided Wilcoxon signed-rank tests were used for ordinal scores. A lower partial score denotes a lower RULA posture category for the assessed region; these partial scores do not constitute a formal RULA grand score or an estimate of overall musculoskeletal risk. Observation-level confidence intervals and *p* values do not account for clustering by surgeon or surgical case and should therefore be interpreted as exploratory. Dashes indicate that mean paired changes were not calculated for ordinal scores.

**Table 4:** Individual paired changes in measured angles.

Pair	Surgeon	Surgical Case ID	Change in Upper Arm Elevation (°)	Change in Elbow Angle (°)	Change in Neck Angle (°)
1	S1	C1	−15.5	+36.2	−5.4
2	S1	C2	−19.9	+30.5	−1.8
3	S1	C3	−11.9	+7.5	+3.7
4	S2	C4	−14.6	+35.3	+10.2
5	S3	C1	−17.9	+47.3	−5.4
6	S4	C2	−6.8	+6.8	+4.9
7	S4	C3	−17.3	+42.9	−4.1
8	S5	C2	−6.6	+19.9	+2.8

**Note:** Changes were calculated as exoscope minus microscope using the unrounded measurements and are presented to one decimal place. Positive values indicate greater angles and negative values indicate smaller angles under exoscopic visualization. Thus, a negative change in upper arm elevation indicates reduced elevation, whereas a positive change in elbow angle indicates greater elbow flexion. Surgeon and surgical case IDs were anonymized. Detailed measurements and RULA-inspired partial scores for each pair are provided in Supplementary Table S2.

**Table 5:** Sensitivity analyses based on surgeon- and surgical-case-level mean changes.

**A. Surgeon-level mean changes**					
**Unit**	**Pair ID(s)**	***n***	**Mean Change in Upper Arm Elevation (°)**	**Mean Change in Elbow Angle (°)**	**Mean Change in Neck Angle (°)**
S1	1, 2, 3	3	−15.8	+24.8	−1.2
S2	4	1	−14.6	+35.3	+10.2
S3	5	1	−17.9	+47.3	−5.4
S4	6, 7	2	−12.0	+24.9	+0.4
S5	8	1	−6.6	+19.9	+2.8
Mean	-	5	−13.4	+30.4	+1.4
**B. Surgical-case-level mean changes.**					
**Unit**	**Pair ID(s)**	***n***	**Mean change in upper arm elevation (°)**	**Mean change in elbow angle (°)**	**Mean change in neck angle (°)**
C1	1, 5	2	−16.7	+41.7	−5.4
C2	2, 6, 8	3	−11.1	+19.1	+2.0
C3	3, 7	2	−14.6	+25.2	−0.2
C4	4	1	−14.6	+35.3	+10.2
Mean	-	4	−14.3	+30.4	+1.7

**Note:** Changes were calculated as exoscope minus microscope using the unrounded measurements and are presented to one decimal place. Negative changes in upper arm elevation indicate reduced elevation, whereas positive changes in elbow angle indicate greater elbow flexion under exoscopic visualization. For each surgeon and surgical case, the change in each angle was averaged across the indicated observation pair(s), so that each surgeon or surgical case contributed one aggregated value to the corresponding sensitivity analysis. In the individual unit rows, *n* denotes the number of observation pairs used to calculate the aggregated value; in each Mean row, *n* denotes the number of aggregated units (five surgeons in panel (A) or four surgical cases in panel (B)). Each Mean row represents the unweighted mean of the aggregated values. Exact two-sided Wilcoxon signed-rank *p* values for upper arm elevation, elbow angle, and neck angle were 0.0625, 0.0625, and 0.8125, respectively, at the surgeon level and 0.1250, 0.1250, and 0.8750, respectively, at the surgical-case level. Given the small numbers of aggregated units, these results should be interpreted descriptively.

## Data Availability

The datasets analyzed in this study are available from the corresponding author upon reasonable request. Public access to the data is restricted to ensure compliance with institutional privacy regulations and to protect patient confidentiality.

## Supplementary Materials

The following supporting information can be downloaded at: https://www.mdpi.com/article/10.3390/brainsci16090954/s1, Table S1: Detailed RULA-inspired partial postural scoring criteria used in this study; Table S2: Individual postural measurements and RULA-inspired partial scores for the eight valid paired observations.
