# Supplementary material for: Exoscope-Associated Upper-Limb Rather than Neck Postural Changes During Frontal Sinus Procedures in Bifrontal Craniotomies
Source: Brain Sci. 2026 Sep 7;16(9):954. doi: 10.3390/brainsci16090954 (PMC13604455; doi:10.3390/brainsci16090954)
Supplement: Supplementary file 1 [file brainsci-16-00954-s001.zip › brainsci-4500307-supplementary.pdf]

Supplementary Materials

Title: Exoscope-Associated Upper-Limb Rather than Neck Postural Changes During Frontal Sinus Procedures in Bifrontal Craniotomies

Table S1. Detailed RULA-inspired partial postural scoring criteria used in this study

Body region	Scoring item	Criterion	Score / additional score	Use in this study / observed status
Upper arm	Basic upper-arm posture	20° extension to 20° flexion	1	Considered; not observed
Upper arm	Basic upper-arm posture	>20° extension or 20°–45° flexion	2	Considered; not observed
Upper arm	Basic upper-arm posture	45°–90° flexion	3	Observed; all analyzed pairs fell into this basic score category
Upper arm	Basic upper-arm posture	≥90° flexion	4	Considered; not observed
Upper arm	Additional upper-arm factor	Shoulder elevation present	+1	Evaluated; observed in 7/8 microscopic images and 0/8 exoscopic images
Upper arm	Additional upper-arm factor	Upper arm abduction present	+1	Evaluated; not observed in the analyzed pairs
Upper arm	Additional upper-arm factor	Arm supported	−1	Considered; not observed / not applied
Forearm	Basic forearm posture	Elbow flexion 60°–100°	1	Evaluated; observed in exoscopic and some microscopic images
Forearm	Basic forearm posture	Elbow flexion <60° or >100°	2	Evaluated; observed especially under microscopic visualization
Forearm	Additional forearm factor	Working across the midline or out to the side, assessed from above	+1	Not applied because the intraoperative photographs were lateral and did not allow reliable overhead assessment

Body region	Scoring item	Criterion	Score / additional score	Use in this study / observed status
Neck	Basic neck posture	Neck flexion 0°–10°	1	Evaluated; observed
Neck	Basic neck posture	Neck flexion >10°–20°	2	Evaluated; observed
Neck	Basic neck posture	Neck flexion >20°	3	Evaluated; observed in a limited number of images
Neck	Basic neck posture	Neck extension	4	Considered; not observed
Neck	Additional neck factor	Neck rotation present	+1	Not applied because neck rotation could not be reliably assessed from the two-dimensional lateral photographs
Neck	Additional neck factor	Neck lateral bending present	+1	Not applied because neck lateral bending could not be reliably assessed from the two-dimensional lateral photographs

Note: The table includes all posture categories considered in the RULA-inspired partial postural assessment, including categories that were evaluated but not observed in the present dataset. The criteria were adapted from the Rapid Upper Limb Assessment (RULA) method for body regions that could be reliably assessed in the intraoperative photographs. A formal RULA total/grand score was not calculated because the full trunk, lower limbs, wrist position, and external load could not be consistently evaluated. These scores were used as supplementary posture indicators.

Table S2. Individual postural measurements and RULA-inspired partial scores for the eight valid paired observations

A. Measured angles

Pair	Surgeon	Surgical case ID	Upper arm elevation (°) Micro	Upper arm elevation (°) Exo	Elbow angle (°) Micro	Elbow angle (°) Exo	Neck angle (°) Micro	Neck angle (°) Exo
1	S1	C1	74.9	59.4	19.5	55.7	17.1	11.7
2	S1	C2	71.9	52.0	42.3	72.9	16.2	14.4
3	S1	C3	56.8	44.9	68.3	75.9	11.8	15.5
4	S2	C4	61.6	47.0	27.1	62.4	16.2	26.5

Pair	Surgeon	Surgical case ID	Upper arm elevation (°) Micro	Upper arm elevation (°) Exo	Elbow angle (°) Micro	Elbow angle (°) Exo	Neck angle (°) Micro	Neck angle (°) Exo
5	S3	C1	75.0	57.1	19.2	66.5	6.6	1.1
6	S4	C2	63.4	56.6	47.1	53.9	10.2	15.1
7	S4	C3	71.6	54.3	26.3	69.3	12.3	8.3
8	S5	C2	63.3	56.7	38.8	58.8	7.9	10.7

B. RULA-inspired partial scores

Pair	Surgeon	Surgical case ID	Upper-arm-related score Micro	Upper-arm-related score Exo	Forearm score Micro	Forearm score Exo	Neck score Micro	Neck score Exo
1	S1	C1	4 (3+1+0)	3 (3+0+0)	2	2	2	2
2	S1	C2	4 (3+1+0)	3 (3+0+0)	2	1	2	2
3	S1	C3	3 (3+0+0)	3 (3+0+0)	1	1	2	2
4	S2	C4	4 (3+1+0)	3 (3+0+0)	2	1	2	3
5	S3	C1	4 (3+1+0)	3 (3+0+0)	2	1	1	1
6	S4	C2	4 (3+1+0)	3 (3+0+0)	2	2	2	2
7	S4	C3	4 (3+1+0)	3 (3+0+0)	2	1	2	1
8	S5	C2	4 (3+1+0)	3 (3+0+0)	2	2	1	2

Note: Values are shown for the eight valid paired observations included in the main analysis. Angular values represent the initial ImageJ measurements used in the main analysis and are presented in degrees to one decimal place. Because the paired changes reported in the main manuscript were calculated from unrounded measurements, a change calculated from the displayed values may differ by 0.1° from the corresponding value in the main manuscript. Surgeon and surgical case IDs were anonymized. Micro, microscope; Exo, exoscope. The upper-arm-related score is presented as the total score, with its components shown in parentheses (basic upper-arm score + shoulder-elevation score + abduction score). A formal RULA total/grand score was not calculated because the full trunk, lower limbs, wrist position, muscle use, and external load could not be consistently assessed.
